# Topoisomerase IIβ Selectively Regulates Motor Neuron Identity and Peripheral Connectivity through Hox/Pbx-Dependent Transcriptional Programs

**DOI:** 10.1523/ENEURO.0404-17.2017

**Published:** 2017-12-14

**Authors:** Michaela Edmond, Olivia Hanley, Polyxeni Philippidou

**Affiliations:** 1 Department of Neurosciences, Case Western Reserve University School of Medicine, Cleveland, OH 44106; 2 Department of Neuroscience and Physiology, Neuroscience Institute, NYU School of Medicine, New York, NY 10016

**Keywords:** Hox genes, motor neurons, Pbx genes, phrenic motor column, topoisomerase IIβ

## Abstract

Vital motor functions, such as respiration and locomotion, rely on the ability of spinal motor neurons (MNs) to acquire stereotypical positions in the ventral spinal cord and to project with high precision to their peripheral targets. These key properties of MNs emerge during development through transcriptional programs that dictate their subtype identity and connectivity; however, the molecular mechanisms that establish the transcriptional landscape necessary for MN specification are not fully understood. Here, we show that the enzyme topoisomerase IIβ (Top2β) controls MN migration and connectivity. Surprisingly, Top2β is not required for MN generation or survival but has a selective role in columnar specification. In the absence of *Top2β*, phrenic MN identity is eroded, while other motor columns are partially preserved but fail to cluster to their proper position. In *Top2β*-/- mice, peripheral connectivity is impaired as MNs exhibit a profound deficit in terminal branching. These defects likely result from the insufficient activation of Hox/Pbx-dependent transcriptional programs as *Hox* and *Pbx* genes are downregulated in the absence of *Top2β*. *Top2β* mutants recapitulate many aspects of *Pbx* mutant mice, such as MN disorganization and defects in medial motor column (MMC) specification. Our findings indicate that *Top2β*, a gene implicated in neurodevelopmental diseases such as autism spectrum disorders, plays a critical, cell-specific role in the assembly of motor circuits.

## Significance Statement

The acquisition of motor neuron (MN) identity is a critical step in the assembly of motor circuits but the molecular pathways underlying MN specification remain unclear. Here, we show that the enzyme topoisomerase IIβ (Top2β) differentially controls MN subtype specification. In the absence of Top2β phrenic MNs do not develop and mice die due to respiratory failure, while other MN subtypes are partially preserved, demonstrating a phrenic-specific critical role for Top2β. We show that Top2β acts via Hox/Pbx-dependent transcriptional programs to control MN organization and medial motor column (MMC) specification, thus unraveling a key step in the genetic hierarchy that underlies MN development.

## Introduction

Topoisomerases are highly conserved enzymes essential for solving topological problems that arise due to the DNA double helical structure during chromosome segregation, DNA replication and transcription (Wang, 2002). Enzymes belonging to the type II family, 2α and 2β, act by introducing double strand DNA breaks and, although they have similar enzymatic activities *in vitro*, have nonoverlapping functions. Topoisomerase IIα (Top2α) is required for cell viability and chromosome segregation while Top2β is dispensable for basic cellular functions and appears to act predominantly within the nervous system (Nitiss, 1998).

Top2β plays a prevalent role during mammalian nervous system maturation (McKinnon, 2016). Mice lacking the *Top2β* gene lack diaphragm motor innervation that results in perinatal lethality due to respiratory failure (Yang et al., 2000). Top2β mutants also exhibit defects in cortical lamination and retinal development (Lyu and Wang, 2003; Li et al., 2014). While the exact mechanism of Top2β action in neurons is not known, it has been implicated in regulating the transcription of subsets of genes during brain development *in vivo* and long genes linked to autism in cultured cortical neurons (Lyu et al., 2006; King et al., 2013). Despite the emerging role of Top2β in the nervous system, it remains unclear whether it has a generic function in neuronal differentiation and survival or whether it exerts unique functions in a cell-specific manner *in vivo* through selective regulation of key downstream targets. Recently, Top2β has been implicated in the transcription of activity-induced immediate early genes suggesting a highly specific and temporally regulated role for this enzyme (Madabhushi et al., 2015). In motor neurons (MNs), Top2β is required for neuromuscular junction (NMJ) formation at the diaphragm muscle. Despite evidence suggesting that Top2β regulates axon growth and neuronal survival (Tiwari et al., 2012; Li et al., 2014), the precise temporal requirement, cell specificity and function of Top2β in MN development remains elusive.

Besides the molecular pathways that underlie NMJ formation, proper innervation of the diaphragm muscle also requires carefully orchestrated transcriptional programs during development that regulate the specification of phrenic motor column (PMC) neurons and their guidance to the diaphragm. The establishment of PMC identity relies on the activity of Hox5 transcription factors (TFs) at cervical levels of the spinal cord (Philippidou et al., 2012). Hox proteins and their cofactors have emerged as critical early determinants of MN identity and connectivity (Philippidou and Dasen, 2013). Along the rostrocaudal axis of the spinal cord, different Hox paralogs control the specification of segmentally-restricted motor columns. At brachial levels a network of Hox proteins determines multiple aspects of lateral motor column (LMC) identity while at thoracic levels, the *Hoxc9* gene is required for the emergence of preganglionic motor column (PGC) neurons (Dasen et al., 2008; Jung et al., 2010). While individual *Hox* genes control the specification of distinct motor columns, they all rely on the activity of Pbx TFs for their functions. *Pbx* genes, members of the three amino acid loop extension (TALE) class of homeodomain proteins, act as cofactors for Hox proteins to establish high affinity binding to transcriptional targets (Merabet and Mann, 2016). In MNs, *Pbx* genes are required for all Hox-dependent specification programs and also play Hox-independent roles in establishing MN organization and topography (Hanley et al., 2016).

The critical functions of Hox/Pbx-dependent programs in MNs rely on a stringent expression pattern during development. The spatial and temporal patterns of *Hox* gene expression in the spinal cord are initially established though morphogen gradients and maintained through Polycomb-dependent repression mechanisms (Liu et al., 2001; Bel-Vialar et al., 2002; Golden and Dasen, 2012). *Pbx* genes also show very specific patterns of expression along the rostrocaudal axis of the spinal cord, as well as elevated expression in certain subsets of MNs (Hanley et al., 2016). While the mechanisms that control the boundaries of *Hox* gene expression in the spinal cord are beginning to emerge, it is less clear how high levels of *Hox* and *Pbx* gene expression are established and maintained in specific populations of MNs.

To understand how Top2β controls NMJ formation and at which level of the transcriptional hierarchy that underlies MN development it acts, we assessed its role in MN specification and peripheral connectivity. Surprisingly, *Top2β* is dispensable for MN generation and survival, but it is differentially required for the emergence of MN columnar identities and proper innervation of peripheral muscles. In the absence of *Top2β*, PMC molecular identity is lost and the majority of phrenic axons do not reach the diaphragm, while other columnar subtypes are partially preserved. We also show that *Top2β* is selectively required for the robust expression of several Hox proteins and the Hox cofactors Pbx1 and Pbx3. Finally, we show that *Top2β* mutants recapitulate many of the phenotypes observed in *Pbx* knockout mice such as defects in medial motor column (MMC) specification. Our results demonstrate that Top2β regulates MN identity in a cell-specific manner by ensuring robust activation of Hox/Pbx-dependent transcriptional programs.

## Materials and Methods

### Mouse genetics

The *Top2β-/-* (Yang et al., 2000) and *Hb9::GFP* (Arber et al., 1999; RRID:IMSR_JAX:005029) lines were generated as described. Mouse colony maintenance and handling was performed in compliance with the protocols approved by the Institutional Animal Care and Use Committee of Case Western Reserve University School of Medicine. Mice were housed in a 12/12 h light/dark cycle in cages containing no more than five animals at a time.

### *In situ* hybridization and immunohistochemistry

*In situ* hybridization and immunohistochemistry were performed as described (Philippidou et al., 2012). Wholemount GFP staining was performed as described (Philippidou et al., 2012) and motor axons were visualized in projections of confocal Z-stacks (500–1000 μm). Wholemounts of diaphragm muscles from e18.5 mice were stained as described (Philippidou et al., 2012). Antibodies were generated as described (Tsuchida et al., 1994; Dasen et al., 2005; Dasen et al., 2008). Other antibodies were used as follows: rabbit anti-Top2β (1:500; Santa Cruz Biotechnology, RRID:AB_2205866), rabbit anti-cleaved Caspase 3 (1:1000; Cell Signaling, RRID:AB_2341188), rabbit anti-Pbx1 (1:2500; Cell Signaling, RRID:AB_2160295), mouse anti-olig2-A488-conjugated (1:500; Millipore, RRID:AB_11205039), rabbit anti-GFP (1:1000; Invitrogen, RRID:AB_221570), goat anti-Scip (1:5000; Santa Cruz Biotechnology, RRID:AB_2268536), rabbit anti-Neurofilament (1:1000; Synaptic Systems, RRID:AB_887743), rabbit anti-Mecom (1:1000; Santa Cruz Biotechnology, RRID:AB_783296), and a-bungarotoxin, Alexa Fluor 555 conjugate (1:1000; Invitrogen, RRID:AB_2617152) . Images were obtained with a Zeiss (LSM 800) confocal microscope or a Zeiss Imager Z2 epifluorescent microscope with Apotome and analyzed with Zen Blue and ImageJ (Fiji).

### Experimental design and statistical analysis

For all experiments a minimum of three embryos per genotype, both male and female, were used for all reported results. In experiments where MN numbers are reported, cells were counted as MNs if they expressed Isl1/2 and as LMC neurons if they expressed FoxP1. Counts were performed in the rostral brachial spinal cord, at the same rostrocaudal levels for both control and mutant animals. The MN numbers reported are either the average (Isl1/2+, FoxP1+) or the total (Scip+) of three to four consecutive sections separated by 160 μm. For the quantitation of Scip, FoxP1, Hox, and Pbx protein levels, the fluorescence intensity in MNs in the ventral spinal cord along three to four consecutive sections was measured in ImageJ (Fiji) and the average fluorescence for each protein was then calculated. Average fluorescent intensity for control protein levels was set to 1, and protein levels in the mutant were expressed as a fraction of control levels. P-values were calculated using paired, two-tailed Student’s *t* test.

## Results

### Expression of Top2β during MN genesis

**Figure 1.**
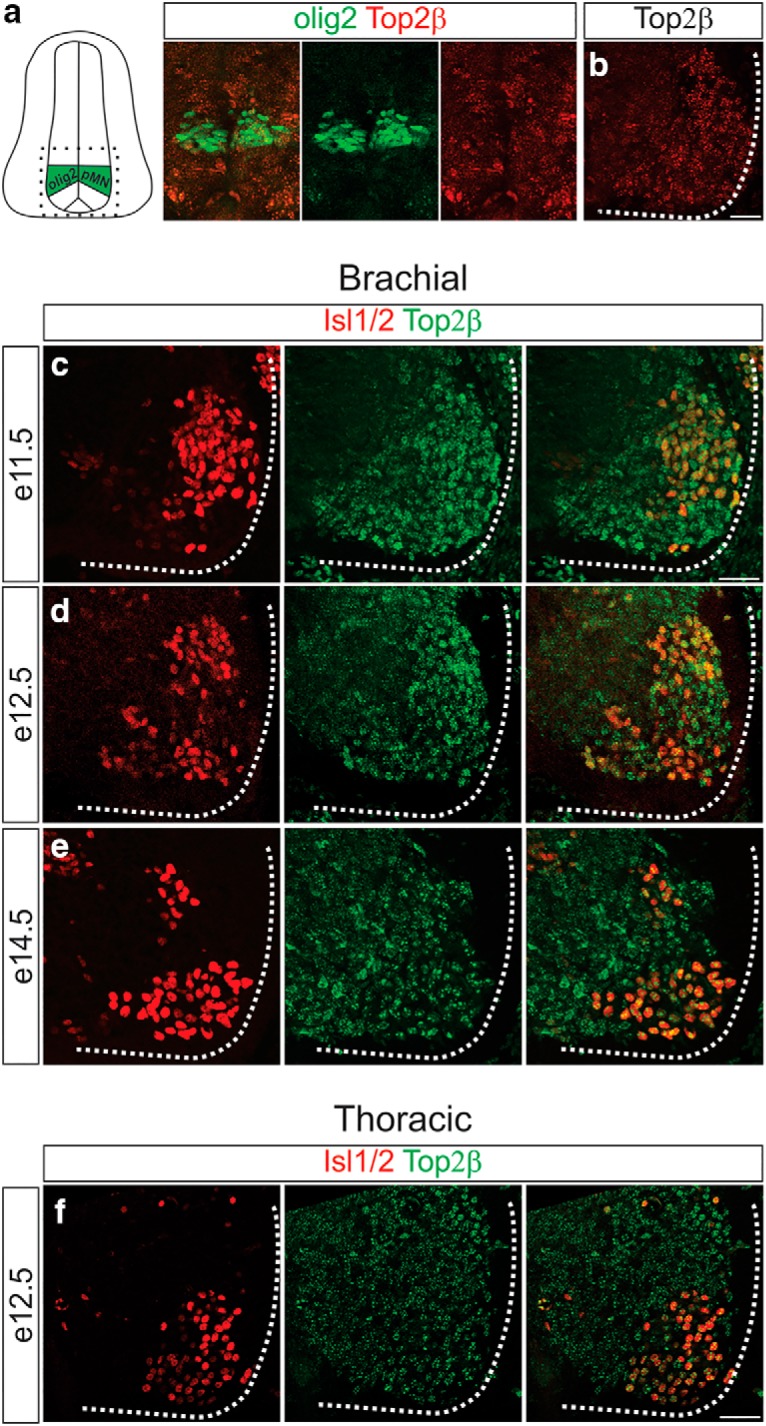
Top2β is expressed at high levels in postmitotic MNs. ***A***, ***B***, Low Top2β expression in MN progenitors (olig2+) at e11.5 (***A***) as compared to the lateral spinal cord where postmitotic MNs are located (***B***). ***C–E***, Top2β is upregulated in postmitotic MNs at brachial levels at e11.5 (***C***), continues to be expressed as MNs navigate to their targets (e12.5; ***D***) and high levels of expression are maintained until later stages of MN development (e14.5; ***E***). ***F***, Top2β expression in MNs at e12.5 at thoracic spinal cord levels. Scale bar = 50 μm.

To establish the temporal requirement for Top2β in MN development we examined the time course of Top2β protein expression in mouse embryonic spinal cord. Consistent with a role of Top2β in postmitotic neurons, expression of Top2β is markedly elevated as MNs exit the cell cycle and begin to migrate. Top2β expression is detected at low levels in the olig2^+^ MN progenitor domain and increases as MNs differentiate and move away from the ventricular zone (Fig. 1*A*,*B*). At embryonic day 11.5 (e11.5), a time when the majority of MNs have already exited the cell cycle (De Marco Garcia and Jessell, 2008), there are increased levels of Top2β expression in the ventral spinal cord coinciding with Isl1/2 expression, a marker for postmitotic MNs, as compared to surrounding non-MN cells (Fig. 1*B*,*C*). Elevated Top2β expression is uniform along rostrocaudal levels of the spinal cord and is maintained, both in brachial and thoracic MNs, as MN axons navigate to the periphery (Fig. 1*D*,*F*). Top2β expression persists in all Isl1/2+ MNs at late embryonic stages (e14.5), after most motor axons have already reached their final targets and initiated branching (Fig. 1*C*).

### Top2β is dispensable for MN generation and survival

**Figure 2.**
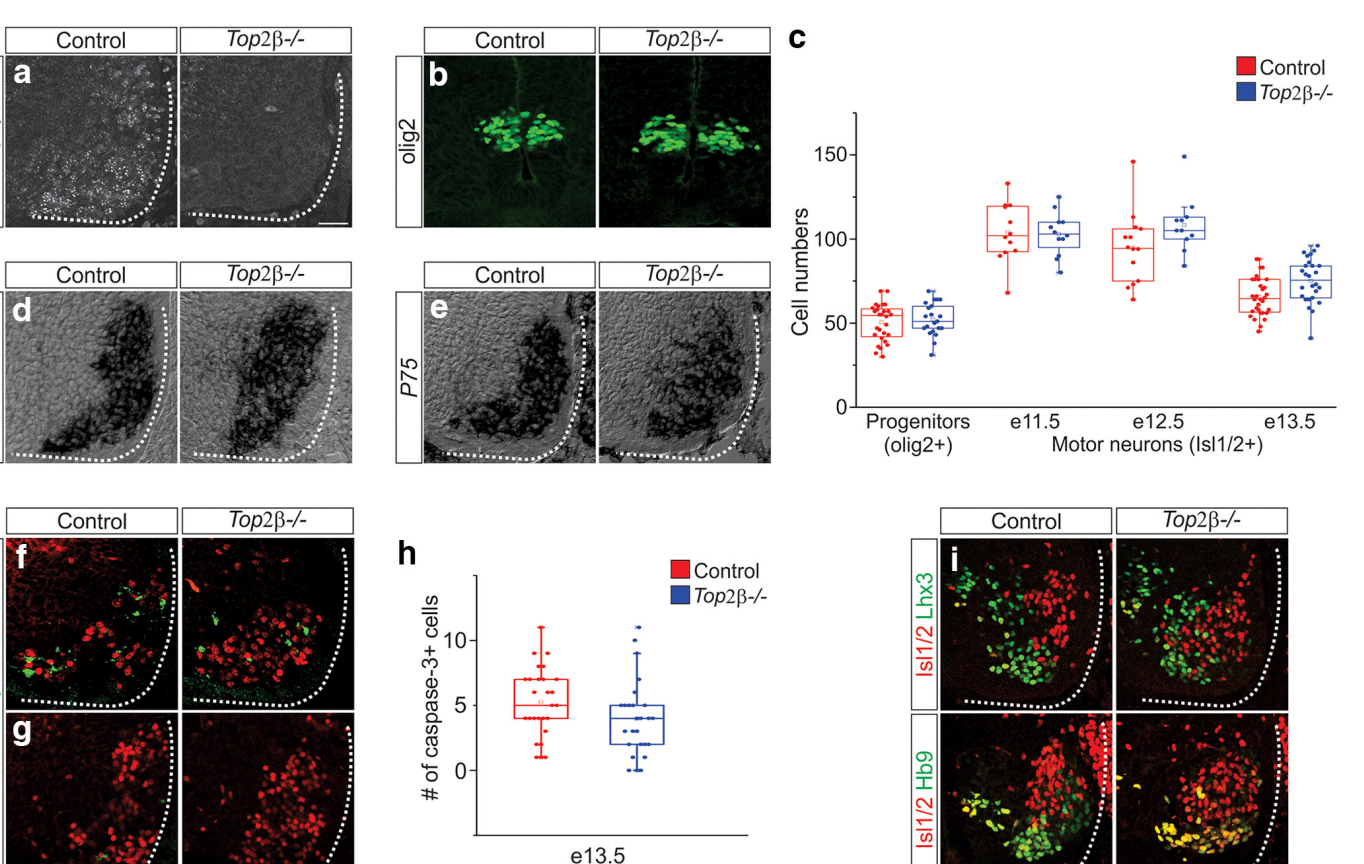
Top2β is not required for MN generation or survival. ***A***, Top2β protein is not detected in *Top2β-/-* mice. ***B***, MN progenitors are unchanged in *Top2β-/-* mice at e11.5, as seen by staining against the MN progenitor marker olig2. ***C***, Quantitation of MN progenitors (olig2+; *p* = 0.27) and postmitotic MNs (Isl1/2+) at brachial levels of the spinal cord (C3-C8) in control and *Top2β-/-*mice at various time points during development (e11.5; *p* = 0.87, e12.5; *p* = 0.2, e13.5; *p* = 0.02). ***D***, *In situ* hybridization against *Vacht* reveals similar numbers of cholinergic MNs in control and *Top2β-/-* mice at e12.5. ***E***, Similar levels of p75 expression in control and *Top2β* mutant mice at e13.5. ***F***, ***G***, Activated caspase 3 staining at e13.5 (brachial levels; ***F***) and e12.5 (thoracic levels; ***G***) does not show an increase in apoptotic cells in *Top2β-/-* mice. ***H***, Quantitation of apoptotic cells in control and *Top2β* mutant mice at e13.5 (*p* = 0.1). ***I***, Staining against the TFs Lhx3, Isl1/2, and Hb9 that collectively mark all MNs demonstrates a disorganization of MN populations in *Top2β-/-* mice at e11.5. Scale bar = 50 μm.

*Top2β* mutant mice show lack of diaphragm innervation and die at birth due to respiratory failure, but the mechanisms of Top2β action in MNs are not known (Yang et al., 2000). To determine the role of Top2β in MN development, we analyzed mice lacking *Top2β*. We confirmed lack of Top2β expression by antibody staining in the spinal cord (Fig. 2*A*). Consistent with low levels of Top2β expression in progenitor cells in the spinal cord, we found that the number and location of MN progenitors in *Top2β-/-* mice at e11.5 was similar to control embryos as assessed by expression of the TF Olig2 (Fig. 2*B*). Since Top2β has been implicated in neuronal survival (Tiwari et al., 2012; Li et al., 2014), we initially compared MN numbers in control and *Top2β-/-* mice. We counted Isl1/2+-expressing MNs in the ventral spinal cord at brachial levels (C3-C8) at multiple embryonic stages and we did not observe a reduction in MN numbers in *Top2β-/-* mice (Fig. 2*C*). Expression of vesicular acetylcholine transporter (*Vacht*), as detected by *in situ* hybridization, also revealed similar numbers of cholinergic MNs in control and *Top2β* mutant mice (Fig. 2*D*). In neurons cultured from *Top2β-/-* mutants, upregulation of the neurotrophin receptor p75 has been associated with premature cell death (Tiwari et al., 2012). We therefore compared levels of p75 expression in MNs in control and *Top2β-/-* mice and saw no significant differences, suggesting that the effects of *Top2β* deletion on gene expression and neuronal phenotype are likely to be cell-type dependent (Fig. 2*E*). In addition, we did not detect an increase in apoptotic cells containing activated caspase 3 in the brachial and thoracic spinal cord of *Top2β-/-* mice at any stage examined (Fig. 2*F–H*). Our results demonstrate that Top2β is not required for MN generation or survival and that the perinatal lethality and MN defects seen in *Top2β-/-* mice are not due to MN cell death.

To further test that initial specification of MNs proceeds normally in the absence of Top2β, we examined the expression of the early postmitotic TFs Lhx3, Isl1/2, and Hb9, which collectively label the entire MN population. Expression patterns of these TFs were similar in control and *Top2β-/-* embryos at e11.5, indicating that dorsoventral patterning and signaling pathways that dictate generic aspects of MN identity are unperturbed. However, we noticed that MNs expressing these markers did not occupy their stereotypical positions in *Top2β-/-* mice (Fig. 2*I*). The inability of MNs to adopt their correct position could reflect defects in their subtype specification. Therefore, we examined whether MNs acquire their correct subtype identities in the absence of Top2β. Since *Top2β-/-* mice die from respiratory failure due to lack of diaphragm innervation (Yang et al., 2000), we first examined whether they exhibit defects in PMC specification.

### Erosion of PMC identity in *Top2β-/-* mice

**Figure 3.**
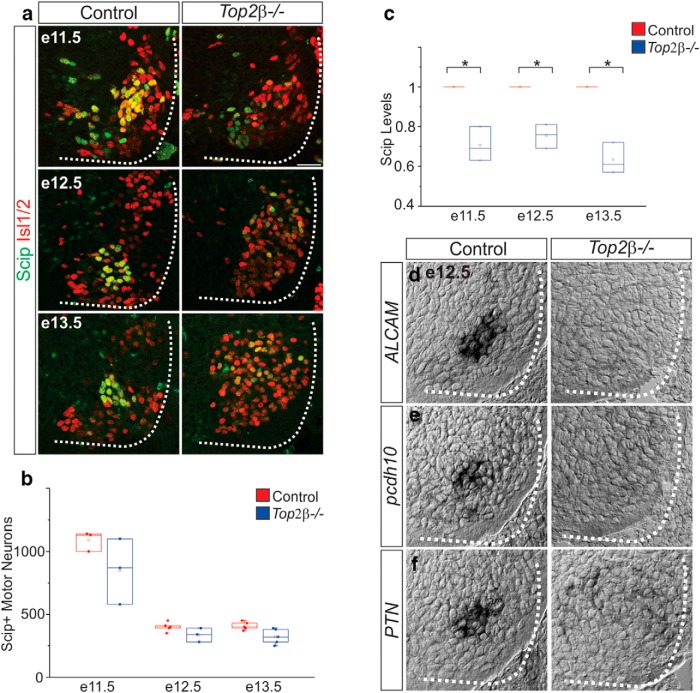
Erosion of PMC identity in *Top2β-/-* mice. ***A***, Scip and Isl1/2 expression in the cervical spinal cord in control and *Top2β-/-* mice at various time points during development. Scale bar = 50 μm. ***B***, ***C***, Quantitation of Scip-expressing MNs (***B***) and Scip protein levels (***C***) in the cervical spinal cord in control and *Top2β-/-* mice (see Materials and Methods for quantitation methods). ***D–F***, Expression of PMC-specific genes *ALCAM* (***D***), *pcdh10* (***E***), and *PTN* (***F***) is abolished in *Top2β-/-* embryos at e12.5.

To determine whether phrenic MN specification is impaired in *Top2β-/-* mice, we examined the expression and distribution of the POU domain TF Scip, which marks PMC neurons at rostral cervical levels. At e11.5, MNs expressing high levels of Scip can be readily seen in the cervical spinal cord of both control and *Top2β-/-* embryos. However, while these MNs are beginning to cluster in control embryos, they are dispersed in *Top2β-/-* mice (Fig. 3*A*). At later embryonic stages scattered Scip+ MNs persist in *Top2β-/-* embryos; however, they are randomly distributed and express lower levels of Scip than phrenic MNs in control animals (Fig. 3*A–C*). To determine whether the lack of clustered, highly expressing Scip MNs reflects the loss of bona fide phrenic MNs, we examined the expression of several known phrenic MN markers (Philippidou et al., 2012; Machado et al., 2014). In *Top2β-/-* mice we observed a complete loss of *ALCAM*, *pcdh10*, and *PTN* expression from the cervical spinal cord at e12.5, indicating that the specification of phrenic MNs is disrupted (Fig. 3*D–F*).

**Figure 4.**
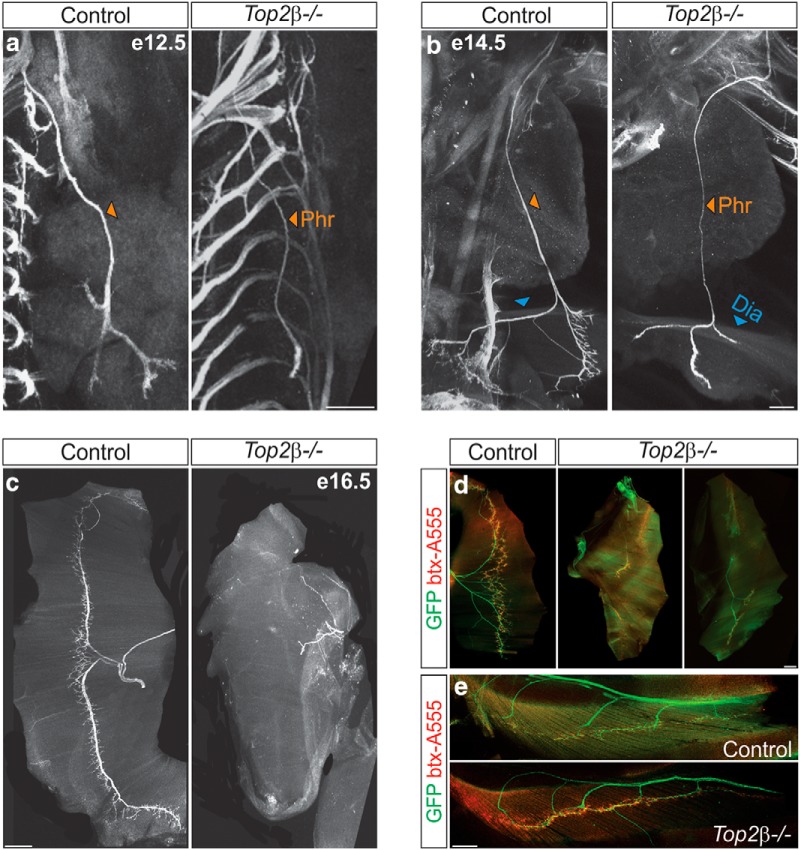
Defects in PMC peripheral connectivity in *Top2β-/-* mice. ***A***, ***B***, Wholemount staining of *Top2β-/-;Hb9::GFP* embryos at e12.5 and e14.5 shows that the phrenic nerve is thinner in mutant mice and does not branch at the diaphragm muscle as compared to control animals. Phr, phrenic nerve; Dia, diaphragm muscle. Scale bar = 200 μm. ***C***, Lack of diaphragm innervation in *Top2β-/-* embryos at e16.5. Scale bar = 500 μm. ***D***, Lack of diaphragm innervation in *Top2β-/-* embryos (right panels) as compared to controls (left panel) at e18.5. Motor axons that reach the diaphragm in *Top2β-/-* embryos initiate synapse formation and cluster postsynaptic acetylcholine receptors. Scale bar = 500 μm. ***E***, Intercostal muscles show normal innervation in *Top2β-/-* mice. Btx-A555, bungarotoxin-Alexa Fluor 555. Scale bar = 200 μm.

To determine whether these changes at the level of the spinal cord lead to alterations in PMC peripheral projections, we examined the trajectory of phrenic axons by wholemount embryo staining. We crossed *Hb9::GFP* mice, expressing GFP in all MNs, to *Top2β* mutants to visualize MN axons. We observed a dramatic decrease in both the thickness of the phrenic nerve and terminal branching in *Top2β-/-* embryos at e12.5 and e14.5 (Fig. 4*A*,*B*). Wholemount muscle staining at e16.5 confirmed the lack of diaphragm innervation in *Top2β-/-* mice as previously reported (Fig. 4*C*; Yang et al., 2000). This defect is likely to be a consequence of the loss of PMC identity, rather than a change in the capacity of MNs to form NMJs. Consistent with this hypothesis, axons that reach the diaphragm in *Top2β-/-* embryos are able to initiate synapse formation and acetylcholine receptor clustering (Fig. 4*D*). Similarly, intercostal muscles that also participate in respiration are normally innervated in *Top2β-/-* mice (Fig. 4*E*), indicating that mutation of *Top2β* has distinct effects on PMC identity. Our data suggest that lack of *Top2β* leads to changes in the specification of distinct MN subtypes, rather than affecting features common to all MNs. To test this hypothesis, we next examined the specification of limb-innervating LMC neurons in *Top2β-/-* mice.

### Defects in MN columnar and pool identities and peripheral connectivity in *Top2β* mutants

**Figure 5.**
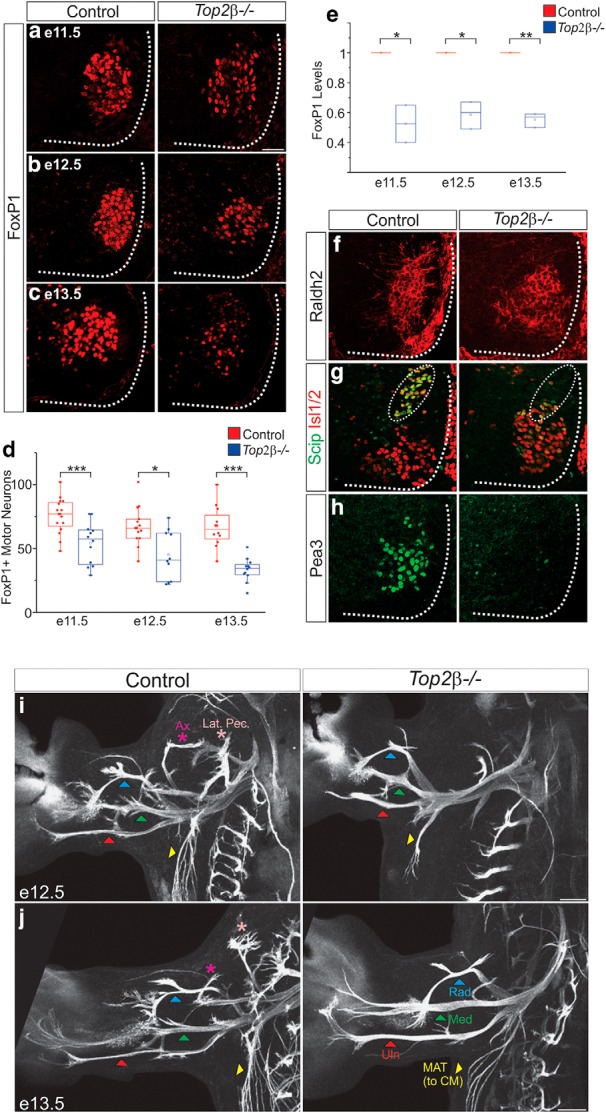
Defects in LMC columnar, pool identities, and peripheral projections in *Top2β-/-* mice. ***A-C***, Reduction in FoxP1 levels in the brachial spinal cord of *Top2β-/-* embryos at e11.5 (***A***), e12.5 (***B***), and e13.5 (***C***). Scale bar = 50 μm. ***D***, Quantitation of FoxP1-expressing MNs in the brachial spinal cord in control and *Top2β-/-* mice at various time points during development (e11.5; *p* = 0.0002, e12.5; *p* = 0.012, e13.5; *p* = 2.85 × 10^−5^). ***E***, Quantitation of FoxP1 protein levels in the brachial spinal cord in control and *Top2β-/-* mice (e11.5; *p* = 0.02, e12.5; *p* = 0.01, e13.5; *p* = 0.004). ***F***, Expression of Raldh2 persists but is diffuse in *Top2β-/-* embryos at e12.5. ***G***, ***H***, The expression of pool-specific markers Scip (***G***) and Pea3 (***H***) is eliminated in *Top2β-/-* embryos at e12.5. ***I***, ***J***, Limb MNs project to the periphery but stay along the three major nerve branches and fail to innervate individual muscles in *Top2β-/-* mice at e12.5 (***I***) and e13.5 (***J***). Projections along proximal nerves are lost in the mutants. Ax, axillary; Lat. Pec., lateral pectoral; Rad, radial; Med, median; Uln, ulnar; MAT, medial anterior thoracic (projecting to CM muscle). Scale bar = 200 μm.

A critical step in the specification of LMC neurons at brachial and lumbar levels of the spinal cord is the induction of the TF FoxP1. We therefore examined the expression profile of FoxP1 in the brachial spinal cord at different developmental time points. Both the number of FoxP1-expressing cells and the levels of FoxP1 protein in MNs are reduced in *Top2β* mutants (Fig. 5*A–E*). Interestingly, unlike the disorganization of PMC neurons, the domain of expression of FoxP1 appears to be initially similar between control and *Top2β* mutant embryos as low-expressing FoxP1 neurons occupy the stereotypical LMC position. This raises the possibility that distinct MN columns are differentially impacted in *Top2β* mutants.

At limb levels, FoxP1 in LMC neurons induces the expression of the retinoic acid (RA) synthetic enzyme *Raldh2.* Despite low FoxP1 levels in *Top2β* mutants, we still observe Raldh2 induction, although at lower levels and more diffusely in the ventral spinal cord (Fig. 5*F*). This further supports the idea that unlike PMC neurons, some features of LMC identity are preserved. To further examine LMC specification we investigated whether the specification of MN pools, clustered groups of MNs innervating the same muscle targets, was affected in *Top2β* mutants. At brachial levels of the spinal cord MNs projecting along the median and ulnar nerves express high levels of the TF Scip while MNs projecting to the cutaneous maximus (CM) and latissimus dorsi (LD) muscles express the TF Pea3. In *Top2β* mutant mice both Scip and Pea3 are dramatically downregulated and the remaining MNs expressing either TF are no longer clustered (Fig. 5*G*,*H*). Our results indicate that while brachial LMC neurons retain some features of their columnar identity, pool identities are more severely affected in *Top2β* mutant mice.

To assess the effects of the *Top2β* mutation on MN axonal trajectories, we examined limb innervation by wholemount immunofluorescence. In *FoxP1-/-* mice, peripheral patterns of innervation are preserved despite the erosion of MN identity and the absence of pool marker expression (Dasen et al., 2008). In *Top2β-/-* mice however we observed distinct changes to peripheral innervation patterns. Consistent with a loss of Pea3, we found a dramatic reduction in CM innervation (Fig. 5*I*,*J*). In contrast, and despite the loss of Scip expression, we surprisingly saw a 70% increase in the thickness of both the ulnar and median nerves in *Top2β-/-* mice at e12.5, accompanied with a loss of branching at target muscles. Similar changes were also seen for the radial nerve (51% increase) indicating that the three major limb-innervating motor nerves are impacted in a similar manner. The increase in nerve thickness is likely a result of a rerouting of all MNs along these 3 major tracts. Consistent with this idea we observe a loss of proximal motor nerves, such as the axillary and lateral pectoral nerves, in *Top2β-/-* mice (Fig. 5*I*,*J*). Our data suggest that in the absence of motor pool specification programs MNs revert to a rudimentary projection pattern along major nerve tracts.

**Figure 6.**
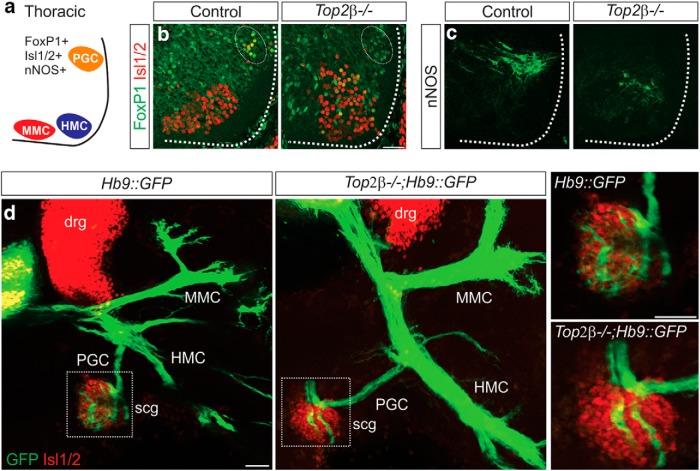
Defects in PGC columnar identities and peripheral projections in *Top2β-/-* mice. ***A***, Organization of motor columns at thoracic levels of the spinal cord; PGC, preganglionic motor column, MMC, medial motor column, HMC, hypaxial motor column. ***B***, ***C***, Expression of PGC markers FoxP1 (white circle; ***B***) and nNOS (***C***) is dramatically reduced in *Top2β-/-* embryos. The remaining neurons expressing nNOS are displaced ventrally. Scale bar = 50 μm. ***D***, PGC neurons project to scg in *Top2β-/-*mice but show an aberrant innervation pattern at the target. scg: sympathetic chain ganglia and drg: dorsal root ganglia. Scale bar = 50 μm.

Since all MNs express high levels of Top2β, we next tested whether columnar specification is impaired at all levels of the spinal cord in *Top2β-/-* mice. At thoracic levels of the spinal cord, PGC neurons projecting to sympathetic chain ganglia (scg) are defined by low levels of FoxP1 and neuronal nitric oxide synthase (nNOS) expression (Fig. 6*A*). In *Top2β* mutants, PGC FoxP1^+^ neurons are not found in their typical position and nNOS expression is greatly reduced (Fig. 6*B*,*C*). The remaining nNOS-expressing neurons are displaced in more ventral positions in the spinal cord. Our results demonstrate that multiple columnar specification programs are affected in *Top2β-/-* mice.

At thoracic levels of the spinal cord PGC neurons innervate neurons along the sympathetic chain (scg). Despite a decrease in the expression of PGC markers and displacement of their cell bodies in *Top2β-/-* mice, projections to the scg appear to be preserved, although there appears to be a defect in the arborization of PGC axon terminals (Fig. 6*D*). These data demonstrate that *Top2β* deletion differentially impacts the connectivity of PGC, LMC, and PMC neurons.

### Top2β is required for the induction and maintenance of high Hox and Pbx protein levels

**Figure 7.**
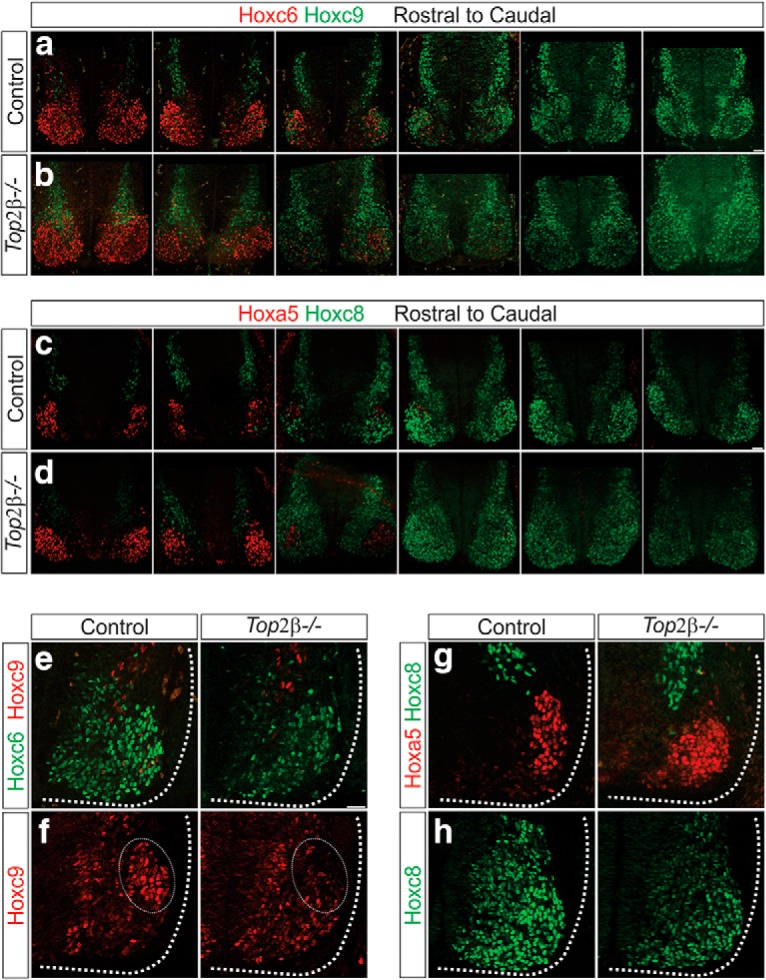
Reduction of Hox protein levels in *Top2β-/-* mice. ***A–D***, The rostrocaudal expression pattern of *Hox* genes is preserved in *Top2β-/-* mice at e11.5. Scale bar = 50 μm. ***E–H***, Hox protein expression levels in control and *Top2β-/-* mice at e11.5. Levels of Hoxc6 (*p* = 0.007), Hoxc8 (*p* = 2.4 × 10^−5^), and Hoxc9 (*p* = 0.04) are decreased while Hoxa5 levels (*p* = 0.84) are unchanged. Scale bar = 50 μm.

**Figure 8.**
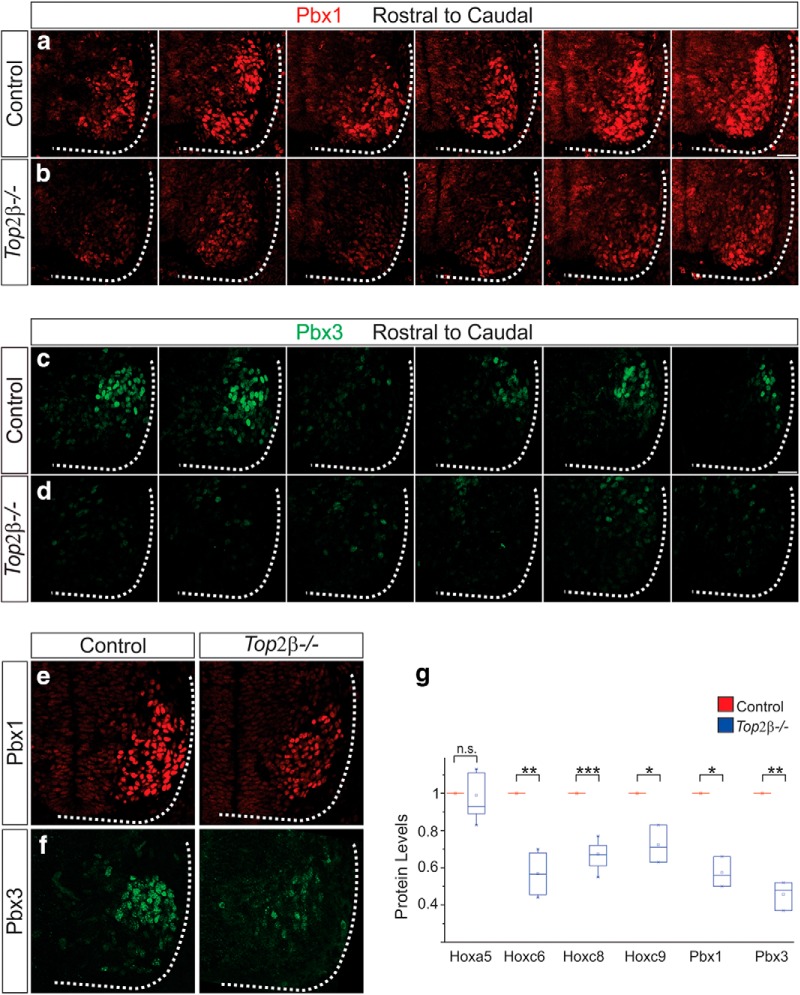
Reduction of Pbx protein levels in *Top2β-/-* mice. ***A–F***, Pbx protein expression levels in control and *Top2β-/-* mice at e11.5. Levels of Pbx1 (*p* = 0.01) and Pbx3 (*p* = 0.007) are decreased at all rostrocaudal levels of the spinal cord. ***G***, Quantitation of Hox and Pbx protein levels at e11.5.

How does Top2β activity contribute to the subtype specification of spinal MNs? Since the acquisition of both columnar and pool identities requires the activity of Hox TFs along the rostrocaudal axis, we examined whether removal of *Top2β* affects Hox and Hox cofactor protein expression. We first assessed the expression of Hox proteins known to have an instrumental role in MN columnar specification: Hoxa5 for PMC, Hoxc6, and Hoxc8 for brachial LMC and Hoxc9 for PGC. In the absence of *Top2β*, the rostrocaudal boundaries of Hox protein expression are preserved, indicating that MN defects are not due to altered Hox protein spatial distribution (Fig. 7*A–D*). We did however observe variable differences in the levels of expression of each Hox protein at e11.5; Hoxc6 and Hoxc8 were attenuated in the brachial spinal cord and high Hoxc9 expression was not seen in presumptive PGC neurons, suggesting that low levels of Hoxc9 may contribute to misspecification of these neurons as previously described (Figs. 7*E–H*, 8*G* ; Jung et al., 2010). Low levels of Hoxc6 and Hoxc8 likely contribute to the columnar and pool specification defects seen in *Top2β-/-* mice. Surprisingly, levels of Hoxa5, the major Hox determinant of PMC identity, were unchanged indicating that the absence of phrenic MNs is not a result of decreased Hoxa5 activity and that Top2β potentially regulates additional PMC determinants.

Pbx1 and Pbx3 are critical cofactors for Hox proteins and are required for PMC specification (Hanley et al., 2016). In *Top2β* mutants, we find that levels of both Pbx1 and Pbx3 are greatly reduced at e11.5 and this loss was observed at all rostrocaudal levels of the spinal cord (Fig. 8*A–G*). This more widespread downregulation suggests that *Pbx* genes function as downstream effectors of Top2β and that their loss may be responsible for the phenotypes observed in *Top2β-/-* mice.

### Top2β controls MN organization and MMC identity

**Figure 9.**
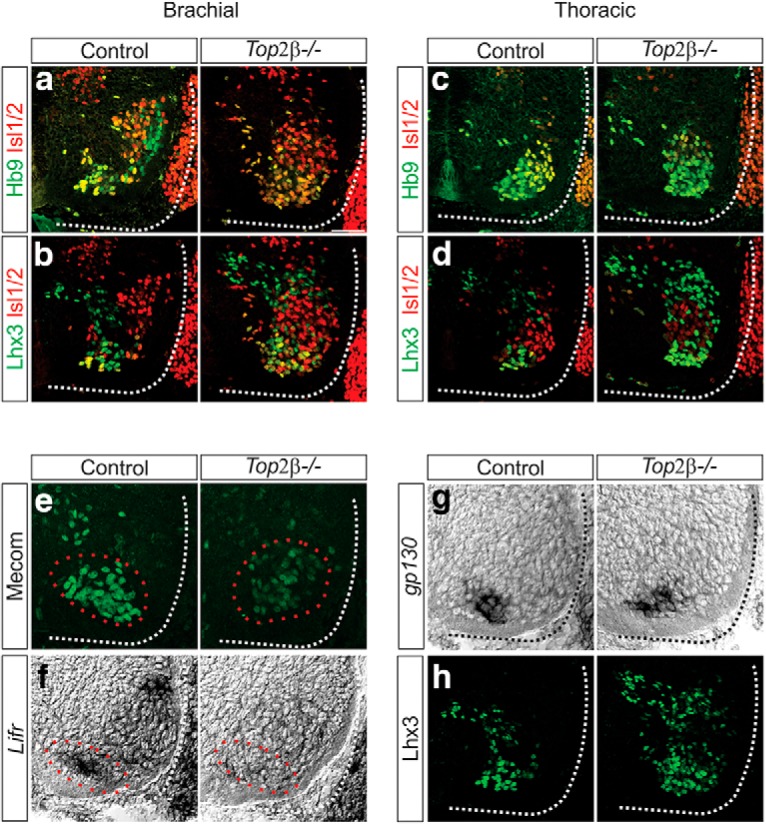
Defects in MN organization and MMC specification in *Top2β-/-* mice. ***A–D***, Disorganization of MNs in *Top2β-/-* mice at e12.5 at brachial (***A***, ***B***) and thoracic (***C***, ***D***) levels of the spinal cord. Hb9+ and Isl1/2+ MNs do not appear to segregate in *Top2β-/-* mice, similar to the phenotype observed in *Pbx1/3* mutant animals. Scale bar = 50 μm. ***E***, ***F***, Effects of *Top2β* deletion on the expression of MMC molecular markers at brachial levels of the spinal cord at e12.5. Mecom and *Lifr* (red circles), both targets of Pbx proteins, are downregulated in *Top2β-/-*mice. ***G***, ***H***, Expression of gp130 (***G***) and Lhx3 (***H***) persist in *Top2β-/-*mice at e12.5. Dorsal Lhx3 expression corresponds to a population of V2 interneurons that appears to be expanded in *Top2β-/-* mice.

To test whether Pbx proteins act downstream of Top2β, we examined whether *Top2β* mutants exhibit additional defects in MN specification that are similar to those observed in *Pbx* mutants. While *Pbx* genes are required for the induction of Hox-dependent MN specification programs, they also have independent roles in MN topography and axial muscle-innervating MMC neuron development. We examined whether MNs in *Top2β* mutants exhibit similar disorganization as *Pbx* mutants. Unlike in control embryos, where MNs expressing Isl1/2 and Hb9 progressively segregate, we found that these MN populations were intermixed both at brachial and thoracic levels in *Top2β-/-* mice (Fig. 9*A–D*). In *Top2β-/-* embryos, Isl1/2+ MNs occupied a more medial position in the spinal cord and failed to migrate to their most dorsal positions, similar to the migration defects observed in Pbx mutants (Hanley et al., 2016).

We next examined whether MMC specification is affected in *Top2β-/-* mice. MMC neurons are found at all rostrocaudal levels of the spinal cord and their development is thought to be Hox-independent. However, MMC-restricted genes are depleted in *Pbx* mutants, indicating that *Pbx* genes play a Hox-independent role in the specification of this population (Hanley et al., 2016). We examined the expression of *Mecom* and *lifr*, two genes that require Pbx proteins for MMC-restricted expression. Both of these genes were found to be downregulated in *Top2β* mutants (Fig. 9*E*,*F*). It is worth noting that not all MMC-specific gene expression is lost in *Top2β* mutants. Both the TF Lhx3 and the cytokine receptor gp130, which are highly enriched in the MMC, persist in Top2β mutants (Fig. 9*G*,*H*; Schaller et al., 2017). Our results indicate that Top2β dictates MN specification through Hox and Pbx-dependent transcriptional programs.

## Discussion

The acquisition of proper MN subtype identity is a critical step in the assembly of motor circuits and the execution of vital functions such as respiration and locomotion. We found that Top2β plays an essential role in MN development, acting through early, subtype-specific transcriptional programs during MN columnar and pool differentiation. We discuss the role of Top2β in MN development and potential mechanisms of Top2β action.

### Specific functions of Top2β in MNs

Type II topoisomerases solve DNA topological problems by transiently creating double strand breaks to relieve torsional stress during DNA processes such as replication and transcription. Top2β has been implicated in nervous system development and multiple neuronal populations are impaired in *Top2β* mutants (Lyu and Wang, 2003; Nevin et al., 2011; Li et al., 2014). In the absence of *Top2β* mice die at birth from respiratory dysfunction due to the lack of synapses at the diaphragm but the mechanisms by which Top2β affects NMJ formation and the exact function of Top2β in MNs had not been resolved (Yang et al., 2000). We demonstrate that *Top2β-/-* phrenic MNs are not impaired in their ability to form synapses as previously thought and that Top2β acts early in postmitotic PMC neurons to establish specification programs. Our study points to potential mechanisms for Top2β involvement in neurodevelopmental disorders, not necessarily at the time of synapse formation but rather early in development as neuronal subtypes are being generated.

Pharmacological inhibition of Top2β in cultured neurons and deletion of *Top2β in vivo* was shown to affect survival and neurite outgrowth in multiple classes of neurons, suggesting that Top2β may control generic pathways active in multiple neuronal populations to promote axon growth and survival (Tiwari et al., 2012). Our data, however, indicate that *Top2β* is not required *in vivo* for MN survival. We do not observe an upregulation of p75 and a resulting increase in apoptosis as has been reported for other neuronal populations, indicating that Top2β plays distinct roles in different neuronal subtypes. In *Top2β* mutants MNs extend their axons to the periphery and grow along major tracts in the limb but subsequently fail to branch to their specific muscles. These defects in peripheral projections appear to be specific for certain classes of MNs; projections along the phrenic nerve are dramatically reduced while LMC neurons project along major nerve tracks in the limb but fail to branch to individual muscles. This specificity argues against a generic function of Top2β in neurite outgrowth, we rather favor the idea that Top2β acts during a critical window in early MN development to orchestrate the specification of MN subtypes. Our results highlight an unexpected specificity for Top2β function and offer support to the idea that Top2β disruption in the nervous system can have subtle, specific effects, manifesting for example as autism spectrum disorders (King et al., 2013), rather than global dysfunction and wide-spread neuronal cell death.

### Top2β in the specification of columnar MN identity

**Figure 10.**
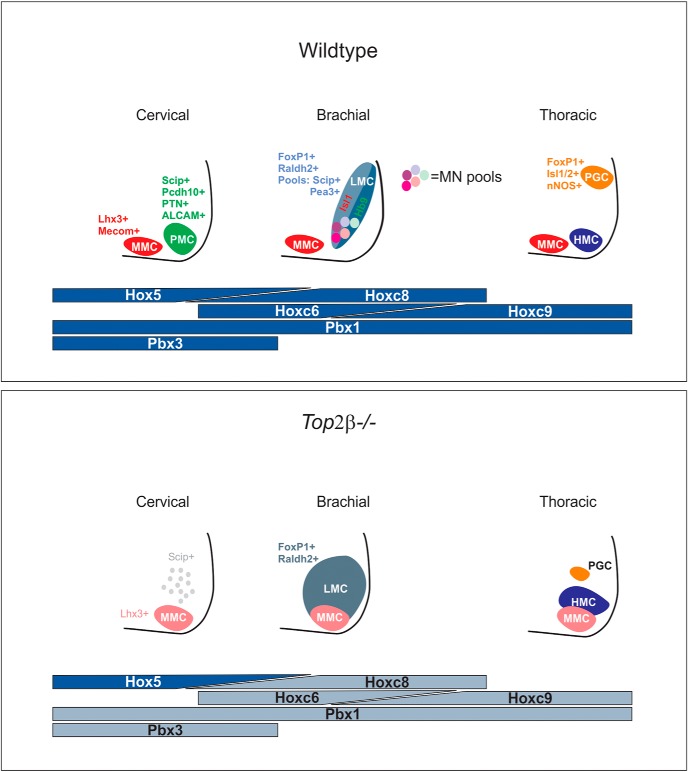
Top2β impacts multiple aspects of MN development through regulation of Hox/Pbx-dependent transcriptional programs. Top2β differentially controls the development of MN subtypes. In the absence of *Top2β*, phrenic MNs are not specified at cervical levels of the spinal cord. At brachial levels, LMC neurons retain aspects of their columnar identity but MN pools are lost. At thoracic levels, PGC neurons become displaced and disorganized. MMC neurons, present at all levels of the spinal cord, lose expression of some of their molecular determinants and are disorganized. These defects largely recapitulate phenotypes observed in *Hox* and *Pbx* mutant mice and the expression of multiple Hox and Pbx proteins is reduced in *Top2β-/-* mice. Our results demonstrate that Top2β functions through the regulation of Hox/Pbx-dependent transcriptional pathways.

Our results indicate that Top2β has a critical role in the specification of MN columnar identity and in the absence of *Top2β* different MN columns are impacted with varying severity (Fig. 10). Phrenic MNs appear to be the most severely affected as *Top2β* mutants do not show induction of any phrenic-specific markers, few axons project along the phrenic nerve and diaphragm innervation is completely absent. While LMC and PGC neurons also appear disorganized, several column-specific markers, such as FoxP1 and nNOS, respectively, are partially preserved. The complete erosion of PMC identity reveals an increased sensitivity of the phrenic MN population to *Top2β* deletion, suggesting the specific regulation of PMC determinants by Top2β. PMC identity is dictated by select transcriptions factors including Hox5 proteins and their cofactors Pbx1 and Pbx3 (Philippidou et al., 2012; Hanley et al., 2016). Our data show that levels of Hoxa5 are not altered in Top2β mutants; however, Pbx1 and Pbx3 protein levels are reduced by ∼50%, suggesting that Pbx downregulation contributes to the defects in PMC specification and connectivity. Consistent with this idea, *Pbx* mutants display defects in PMC specification and diaphragm innervation that are similar to that of *Top2β* mutants.

While *Pbx1/3* and *Top2β* mutants exhibit similar phenotypes in regards to phrenic MNs, the peripheral projections of LMC neurons are differentially affected, with *Top2β* mutants showing increased projections along major nerves while *Pbx1/3* mutants exhibit thinning of most limb-innervating axons. In *Top2β* mutants FoxP1 and Pbx expression is partially preserved, compared to a complete absence in *Pbx1/3* mutants. This difference in the levels of FoxP1 and Pbx likely contributes to the differential effects on LMC connectivity observed. Our data also reveal an increased sensitivity of PMC neurons to fluctuating Pbx levels and a potential dependence of these neurons on high Pbx expression. Both Pbx1 and Pbx3 show highest expression in the cervical spinal cord where phrenic MNs are located when compared to the rest of the spinal cord, suggesting that high levels of the two factors are required in this population. A reduction of Pbx levels by 50% is sufficient to prevent the induction of all PMC markers but only partially affects LMC and PGC induction. Our data underscores that in addition to the temporal and spatial restriction of TF expression, robustness of expression, an often-underappreciated dimension of developmental programs, is also a significant determinant of MN identity.

### Mechanisms of Top2β action

In addition to downregulation of *Hox* and *Pbx* genes and impact on early MN specification programs, the deletion of *Top2β* may also influence the expression of a cohort of MN specific genes involved in later aspects of development such as synaptogenesis. A screen for genes downregulated in cortical neurons in *Top2β* mutants at multiple time points revealed a high number of affected genes, raising the possibility that *Top2β* may have a more global role in transcription throughout development (King et al., 2013). It is also possible that the absence of PMC-specific genes and LMC pool markers such as Pea3 and Scip is a direct result of their transcriptional regulation by Top2β. While we cannot completely rule out these possibilities our data suggest that the defects we observe are mediated through a small set of Top2β target genes rather than a global effect on transcription.

Interestingly, a microarray screen in the embryonic brain identified Pbx1 and Pbx3 as being downregulated in *Top2β* mutant mice, suggesting that a common set of genes is under Top2β regulation that can influence different functions depending on the neuronal cell type (Lyu et al., 2006). The PMC-specific marker ALCAM was also identified in this screen, but unlike its complete absence from phrenic MNs, it was only slightly downregulated in *Top2β-/-* brain, suggesting that the dramatic change we observe likely results from a defect in PMC specification rather than a direct effect of Top2β on *ALCAM* transcription. Consistent with this idea a number of genes that are affected in *Top2β* mutant brains do not exhibit Top2β binding at their promoter regions, indicating that some expression changes are an indirect result of TF downregulation. It has been suggested that the transcription of long genes, including autism-spectrum disorder risk genes, is disproportionately affected in the absence of Top2β activity (King et al., 2013). Pbx1 and Pbx3 are both over 200 kb in length, making them likely direct targets of Top2β. However, we also observe differential effects on Hox protein expression, despite the similar short length of the encoding genes, suggesting that gene length may not be the only determinant for Top2β regulation. Defining the full repertoire of genes affected in *Top2β* mutants and distinguishing between Top2β direct and indirect targets in MNs will further illuminate the role of this protein in MN development.

### Robust transcriptional networks and motor circuit assembly

The complex phenotypes observed in *Top2β* mutants result from a reduction in the expression of key TFs that act during early MN development. Our findings highlight the importance of titrating TF levels during neuronal specification. In MNs for example, robust expression of FoxP1 leads to the acquisition of LMC identity, while low levels of FoxP1 underlie PGC specification (Dasen et al., 2008). The differential effects seen on distinct motor columns in *Top2β* mutants indicate that certain MN subtypes are more sensitive to even small fluctuations in TF expression (Fig. 10). The specification of PMC neurons is dramatically halted in *Top2β-/-* mice indicating that there is a critical requirement for high Pbx expression in the induction of PMC-specific genes. In contrast, LMC-specific Raldh2 expression, which is abolished in Pbx1/3 mutants, persists in *Top2β* mutants, indicating a less stringent requirement for high Pbx levels for the expression of LMC determinants. Our data suggests that the degree of robustness of transcriptional regulatory networks, established through the activity of Top2β, is a critical determinant of neuronal cell identity programs and serves as an additional strategy employed in the nervous system to generate cell diversity. An intriguing possibility is that *Top2β* also acts in multiple interneuron populations in the spinal cord to initiate further subtype specification. In support of this idea, *Top2β-/-* mice show changes in the localization of Lhx3+ interneurons in the spinal cord (Fig. 9*H*). Our findings establish *Top2β* as a critical mediator in the assembly of motor circuits and *Top2β* mutant mice will provide a powerful tool to further dissect the transcriptional pathways that give rise to diverse populations of neurons in the mammalian spinal cord.

## Synthesis

Reviewing Editor: Esther Stoeckli, University of Zurich Decisions are customarily a result of the Reviewing Editor and the peer reviewers coming together and discussing their recommendations until a consensus is reached. When revisions are invited, a fact-based synthesis statement explaining their decision and outlining what is needed to prepare a revision will be listed below.

I have carefully read the manuscript in the context of the reviewers' suggestions to the original version of the manuscript that you added as companion manuscript.

While I think that you generally improved the manuscript according to the comments made by the reviewers of the first version of the manuscript submitted to J.Neurosci. I feel that the Discussion ends too abruptly. I would be good to conclude with a summary of the model added as Figure 10. Also it would be desirable to expand on the importance of the suggested role of Top2β in the context of a general model as suggested by reviewer 2 on the original version of the manuscript (“The findings are relevant to unmask the complex effects of Top2β in motor system development and suggest that the degree of robustness of transcriptional regulatory networks established through the activity of Top2β is a critical determinant of neuronal cell identity programs. This is a concept of general interest that would be worth expanding further in the Discussion.”

In Figure 2c the y axis label is missing.

## Author Response

November 30, 2017

Dear Dr. Stoeckli,

Thank you for considering

our manuscript entitled “Topoisomerase IIβ Selectively Regulates Motor Neuron Identity and Peripheral Connectivity Through Hox/Pbx-dependent Transcriptional Programs” by Edmond et al. for publication in eNeuro. We have made changes to the manuscript according to editorial and reviewer comments and we hope that you will find this current improved version of the manuscript suitable for publication in eNeuro.

Below please find our responses to the synthesis of reviews:

Significance Statement Comments for Author (Required):

OK as is

We have included the Significance Statement within the manuscript as requested by the editorial stuff.

Synthesis Statement for Author (Required):

I have carefully read the manuscript in the context of the reviewers' suggestions to the original version of the manuscript that you added as companion manuscript.

While I think that you generally improved the manuscript according to the comments made by the reviewers of the first version of the manuscript submitted to J.Neurosci. I feel that the Discussion ends too abruptly. I would be good to conclude with a summary of the model added as Figure 10. Also it would be desirable to expand on the importance of the suggested role of Top2β in the context of a general model as suggested by reviewer 2 on the original version of the manuscript (“The findings are relevant to unmask the complex effects of Top2β in motor system development and suggest that the degree of robustness of transcriptional regulatory networks established through the activity of Top2β is a critical determinant of neuronal cell identity programs. This is a concept of general interest that would be worth expanding further in the Discussion.”

We have now included a section in the Discussion entitled: “Robust transcriptional networks and motor circuit assembly” to highlight the role of the robust expression of transcription factors in neuronal development and the assembly of motor circuits.

In Figure 2c the y axis label is missing.

We have included a y-axis label in the figure.

We hope that with these changes our manuscript will be suitable for publication in eNeuro. We have included both a clean copy of the manuscript and one indicating the changes, along with tiff files for individual figures.

Thank you for considering our manuscript for publication in eNeuro.

Sincerely,

Polyxeni Philippidou, PhD
